# Relationship of neutrophil/lymphocyte ratio with cerebral small vessel disease and its common imaging markers

**DOI:** 10.1002/iid3.1228

**Published:** 2024-04-05

**Authors:** Jiangping Cai, Xiaoyi Zeng, Xiaojin Huang, Hansheng Dong, Junyi Liu, Jie Lin, Meirong Xie, Xiaolan Wei

**Affiliations:** ^1^ Department of Neurology The First Hospital of Quanzhou Affiliated to Fujian Medical University Fujian China

**Keywords:** cerebral small vessel disease, enlarged perivascular spaces, inflammation, neutrophil/lymphocyte ratio, white matter hyperintensity

## Abstract

**Background:**

High neutrophil/lymphocyte ratio (NLR) is associated with poor prognosis in ischemic stroke. However, the role of NLR in cerebral small vessel disease (CSVD) is controversial. Herein, we evaluated the value of NLR in identifying CSVD and its relationship with the common imaging markers of CSVD.

**Methods:**

A total of 667 patients were enrolled in this study, including 368 in the CSVD group and 299 in the non‐CSVD group. Clinical, laboratory, and imaging data were collected. The relationship of NLR with CSVD and common imaging markers of CSVD were analyzed with univariate and multivariate logistic regression analysis. The predictive value of NLR was assessed with the receiver operating characteristic curve.

**Results:**

NLR (odds ratio [OR] = 1.929, 95% confidence interval [CI] =  1.599–2.327, *p* < .001) was an independent risk factor for CSVD. NLR was also independently associated with moderate to severe white matter hyperintensity (WMH) (OR = 2.136, 95% CI = 1.768–2.580, *p* < .001), moderate to severe periventricular WMH (OR = 2.138, 95% CI = 1.771–2.579, *p* < .001), and moderate to severe deep WMH (OR = 1.654, 95% CI = 1.438–1.902, *p* < .001), moderately to severely enlarged perivascular spaces (EPVS) (OR = 1.248, 95% CI = 1.110–1.402, *p* < .001), moderately to severely EPVS in the basal ganglia (OR = 1.136, 95% CI = 1.012–1.275, *p* = .030), and moderately to severely EPVS in the centrum semiovale (OR = 1.140, 95% CI = 1.027–1.266, *p* = .014). However, NLR was not statistically significantly associated with lacune. The optimal cutoff point of NLR in predicting CSVD was 2.47, with sensitivity and specificity of 84.2% and 66.9%, respectively (*p* < .01). The diagnostic effect was maximized when NLR was combined with other risk factors.

**Conclusions:**

NLR is an independent risk factor for CSVD and is independently associated with common imaging markers of CSVD. NLR may serve as a valid and convenient biomarker for assessing CSVD.

## Graphical abstract

NLR was significantly related with CSVD and common imaging markers, including WMH, PWMH, DWMH, EPVS, BG‐EPVS, and CSO‐EPVS. NLR may serve as a valid and convenient biomarker for assessing CSVD.

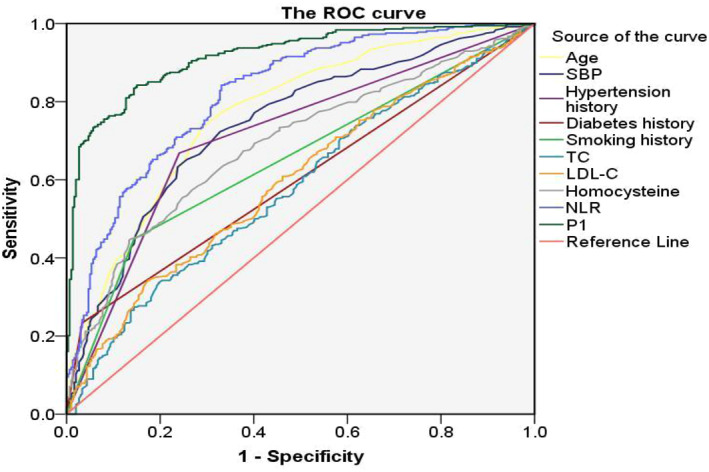

## 1 INTRODUCTION

Cerebral small vessel disease (CSVD) is the most common pathological and neurological syndrome caused by cerebrovascular lesions, which has been considered a main contributor to the occurrence and recurrence of stroke and dementia.^1^ Inflammation is closely related to the development of CSVD.^2^, ^3^, ^4^, ^5^ In detail, systemic inflammation plays important roles in endothelial dysfunction, blood–brain barrier permeability, and cerebral blood flow autoregulation, which may thus affect the development of CSVD.^5^, ^6^, ^7^ Moreover, the close association between biomarkers of inflammation and imaging features of CSVD has been shown.^6^ The imaging features of CSVD mainly include new small subcortical infarcts, vasogenic lacune (Lacune), white matter hyperintensity (WMH), enlarged perivascular space (EPVS), cerebral microbleeds, and encephalatrophy.^2^, ^5^, ^6^, ^7^ One previous study showed that endothelium‐associated inflammatory markers were associated with WMH volume and Lacune, but not with cerebral microbleeds or perivascular spaces.^6^ Mitaki et al. found that higher levels of high‐sensitivity C‐reactive protein were associated with lacunar infarction.^7^ EPVS, which is an extension of fluid‐filled spaces outside the brain that crosses gray and white matter along the course of blood vessels, is also significantly associated with neuroinflammatory biomarkers.^8^

Neutrophil/lymphocyte ratio (NLR), the ratio of neutrophil count to lymphocyte count, has been considered an easily accessible inflammatory marker.^9^ It could reflect the role of neutrophils in nonspecific immune responses, and the role of lymphocytes as key factors in specific immune responses.^10^ The increase of NLR is often caused by the increase of neutrophils and the decrease of lymphocytes, indicating the upregulated innate immunity and the downregulated acquired immunity.^11^ Neutrophils can be recruited to the ischemic areas of brain tissue following stroke, and destroy the blood–brain barrier by releasing proteolytic enzymes or free oxygen radicals and other inflammatory mediators, thus causing secondary brain injury or hemorrhagic transformation.^12^, ^13^ Lymphocytes secrete anti‐inflammatory cytokines, such as interleukin‐4 (IL‐4), IL‐10, and IL‐13, and promote the production of nerve growth factors and remodeling and repair of ischemic brain tissue.^14^, ^15^ NLR is significantly associated with the development of inflammatory diseases, including CSVD.^16^, ^17^, ^18^, ^19^, ^20^, ^21^, ^22^, ^23^, ^24^, ^25^ The existing literature^26^, ^27^ has demonstrated a relationship between the net quantities of neutrophils and lymphocytes and cardiovascular disease. In comparison to individual blood indices like total white blood cell count or neutrophil count, the NLR is deemed to have a better predictive ability for cardiovascular disease.^26^ However, there is a debate concerning the association between NLR and CSVD.^11^, ^28^ Furthermore, no report focuses on the association between NLR and imaging features of CSVD, such as WMH and EPVS at different sites.

Therefore, in this study, we conducted a retrospective study to elucidate the relationship between NLR and CSVD. The relationship between NLR and imaging features of CSVD was further evaluated. The value of NLR as a biomarker for predicting the occurrence of CSVD was determined. Our findings may provide further evidence for understanding the pathogenesis of CSVD.

## 2 METHODS

### 2.1 Ethics

All methods were carried out following the Declaration of Helsinki. This study was approved by the Ethics Committee of Quanzhou First Affiliated Hospital of Fujian Medical University (approval no. [2020]168). Written informed consent was obtained from each participant.

### 2.2 Participants

This is a retrospective study. Patients with CSVD who visited the Department of Neurology, Quanzhou First Affiliated Hospital of Fujian Medical University from July 2017 to December 2021 were recruited. Inclusion criteria: (1) patients aged 36–85 years old; (2) patients underwent cranial magnetic resonance imaging (MRI) examination, including T1‐weighted imaging (T1WI), T2‐weighted imaging (T2WI), fluid‐attenuated inversion recovery (FLAIR) imaging, and diffusion‐weighted imaging (DWI); (3) patients underwent laboratory blood tests for detecting biochemical indicators, such as serum creatinine and homocysteine, during hospitalization. Exclusion criteria: (1) patients with a prior history of large cerebral infarction resulting from the occlusion of large vessels, or other conditions that impeded the diagnosis of CVSD. The diagnosis of large vessel occlusion was determined based on the magnetic resonance angiography or computed tomography angiography examination of the cerebral vessels. A narrowing of 100% in large blood vessels detected during vascular examination can be classified as occlusion. (2) Patients with severe stenosis and occlusion of large vessels in the brain on computed tomography angiography or digital subtraction angiography. (3) Patients who had contraindications (such as cardiac pacemaker placement, heart replacement prosthesis, previous aneurysm surgery, and intracranial metal aneurysm clipping) for MRI imaging examination. (4) Patients with severe heart disease, liver or kidney failure, or a history of stroke. (5) Patients with blood diseases, malignant tumors, or autoimmune diseases. (6) Patients with infection in the past 2 weeks. (7) Patients who were currently under medication with glucocorticoids and immunosuppressive agents. (8) Patients with acute cerebral hemorrhage, acute subarachnoid hemorrhage, or previous history of cerebrovascular malformation or aneurysmal subarachnoid hemorrhage. (9) Patients with neurodegenerative diseases, such as Parkinson's disease, Alzheimer's disease, and so forth. (10) Patients with clear nonvascular white matter lesions, such as multiple sclerosis, adult white matter dysplasia, metabolic encephalopathy, and so forth. (11) Patients with incomplete clinical data.

### 2.3 MRI scans and image analysis

The 3.0T cranial MRI (Signa, GE Healthcare) scans, including T1WI, T2WI, FLAIR, and DWI sequences, were conducted for each enrolled patient. WMH, Lacune, and EPVS were defined as MRI markers of CSVD. WMH was defined as increased brightness on T2 images in the brain white matter. The periventricular WMH (PWMH) and deep WMH (DWMH) were evaluated according to the Fazekas rating scale.^29^ The total WMH burden was calculated as the sum of PWMH and DWMH scores and ranged between 0 and 6 points. According to the severity of the total burden of WMH or the scores of PWMH and DWMH, the study population was divided into mild (WMH scores 0–2 points or PWMH and DWMH scores 0–1 point) and moderate to severe (WMH scores 3–6 points or PWMH and DWMH scores 2–3 points) groups. EPVS was defined as small (<3 mm) punctate or linear hyperintensities on T2 images. The EPVS in basal ganglia (BG‐EPVS) and EPVS in centrum semiovale (CSO‐EPVS) were assessed. The number of EPVS in one hemisphere at the most severely affected level in different brain regions was calculated, and then graded according to the following criteria^30^: 0 = no EPVS, 1 = 1–10 EPVS, 2 = 11–20 EPVS, 3 = 21–40 EPVS, 4 = more than 40 EPVS. According to the severity of EPVS or the scores of BG‐EPVS and CSO‐EPVS, we divided the study population into the mild group (EPVS ≤ 10 or BG‐EPVS and CSO‐EPVS score of 0–1) and the moderate to severe group (EPVS > 10 or BG‐EPVS and CSO‐EPVS score of 2–4).^31^ Lacune was a round or ovoid lesion in cerebrospinal fluid signal with a diameter of 3–20 mm.^32^ The cranial MRI images were assessed by two trained neurologists blinded to baseline data independently. Any discrepancies were resolved by consultation with a neuroimaging specialist.

### 2.4 Demographic and clinical data collection

Baseline demographic and clinical data were collected by trained investigators. The collected baseline data included gender, age, height, weight, smoking history, drinking history, history of hypertension, diabetes, coronary heart disease (CHD), and so forth.

### 2.5 Laboratory assessment

The fasting blood samples were obtained from each participant and were assessed for the following indicators: white blood cell count (WBC), neutrophil count, lymphocyte count, C‐reactive protein (CRP), triglyceride (TG), total cholesterol (TC), low‐density lipoprotein cholesterol (LDL‐C), hemoglobin A1c (HbA1c), homocysteine, fasting plasma glucose (FPG), and uric acid. The estimated glomerular filtration rate (eGFR) was estimated using the Chronic Kidney Disease Epidemiology Collaboration equation. NLR was calculated as the ratio of the absolute neutrophil count to the absolute lymphocyte count.

### 2.6 Statistical analysis

Statistical analysis was performed by using SPSS 23.0 (IBM SPSS) and GraphPad Prism 8 (GraphPad Software Inc.). The missing data were not included in the study. Continuous variables are presented as means ± standard deviations or medians (interquartile ranges) and were assessed using the Kruskal–Wallis test or Mann–Whitney *U* test. Categorical variables are presented as frequencies (percentages) and compared with the chi‐square test or Fisher's exact test. *p* Values <.05 were considered statistically significant.

Variables with *p* < .05 in univariate analysis were included in the multivariate logistic regression model, and odds ratios (ORs) and 95% confidence intervals (CIs) were calculated. In multivariate logistic regression model 1, the age and sex were adjusted. The multivariate logistic regression model 2 was based on model 1 and the factors of body mass index, systolic blood pressure (SBP), diastolic blood pressure (DBP), hypertension history, diabetes history, CHD history, smoking history, drinking history, CRP, FPG, HbA1c, TG, TC, LDL‐C, homocysteine, eGFR, and uric acid were adjusted. The model's fit was assessed using the AIC (Akaike Information Criterion). In addition, receiver operating characteristic (ROC) curves were used to assess the ability of NLR to identify CSVD.

## 3 RESULTS

### 3.1 Baseline characteristics

In this study, a total of 667 patients were finally enrolled, with a median age of 63 (52–70) years. Their baseline data are shown in Table 1. There were significant differences between CSVD and non‐CSVD groups in terms of gender, age, hypertension history, diabetes history, CHD history, smoking history, drinking history, SBP, DBP, neutrophil count, lymphocyte count, CRP, FPG, HbA1c, TC, LDL‐C, homocysteine, eGFR, uric acid, and NLR (all *p* < .05), but there was no significant difference in body mass index, WBC, and TG (all *p* > .05) (Table 1).

**Table 1:** Baseline characteristics.

	CSVD (*n* = 368)	Non‐CSVD (*n* = 299)	*p*
Sex, male, *n* (%)	242 (65.8)	137 (45.8)	<.001
Age (years)	67 (60–73)	54 (47–64)	<.001
BMI (kg/m^2^)	23.60 (21.99–24.74)	23.62 (21.97–25.09)	.596
Hypertension history, *n* (%)	246 (66.8)	72 (24.1)	<.001
Diabetes history, *n* (%)	86 (23.4)	10 (3.3)	<.001
CHD history, *n* (%)	38 (10.3)	5 (1.7)	<.001
Smoking history, *n* (%)	165 (44.8)	43 (14.4)	<.001
Drinking history, *n* (%)	71 (19.3)	14 (4.7)	<.001
SBP, mmHg	145 (132–164)	125 (115–138)	<.001
DBP, mmHg	87 (78–98)	80 (73–90)	<.001
WBC (×10^9^/L)	6.76 (5.62–8.15)	6.79 (5.77–8.38)	.459
Neutrophil count (×10^9^/L)	4.59 (3.62–5.82)	3.99 (3.11–5.50)	<.001
Lymphocyte count (×10^9^/L)	1.21 (0.98–1.58)	1.99 (1.62–2.52)	<.001
CRP, mg/L	2.27 (0.52–5.00)	0.53 (0.50–2.40)	<.001
FPG, mmol/L	5.34 (4.77–6.19)	5.04 (4.65–5.55)	<.001
HbA1c (%)	5.90 (5.60–6.50)	5.70 (5.40–5.95)	<.001
TG, mmol/L	1.24 (0.90–1.82)	1.23 (0.90–1.72)	.753
TC, mmol/L	4.76 (3.91–5.52)	5.07 (4.27–5.92)	<.001
LDL‐C, mmol/L	3.06 (2.37–3.66)	3.34 (2.72–3.92)	<.001
Homocysteine, μmol/L	11.20 (8.60–15.00)	8.60 (6.90–10.60)	<.001
eGFR (mL/min/ 1.73 m^2^)	91.90 (75.77–102.56)	102.82 (91.71–111.09)	<.001
Uric acid, μmol/L	361.00 (292.00–439.00)	325.00 (269.00–396.00)	<.001
NLR	3.54 (2.68–4.95)	2.08 (1.54–2.87)	<.001

*Note*: Data are presented as medians (interquartile ranges) or *n* (%).

Abbreviations: BMI, body mass index; CHD history, coronary heart disease history; CRP, C‐reactive protein; CSVD, cerebral small vessel disease; DBP, diastolic blood pressure; eGFR, estimated glomerular filtration rate; FPG, fasting plasma glucose; HbA 1c, hemoglobin A1c; LDL‐C, low‐density lipoprotein cholesterol; NLR, neutrophil/lymphocyte ratio; SBP, systolic blood pressure; TC, total cholesterol; TG, triglyceride; WBC, white blood cell.

### 3.2 NLR is independently associated with CSVD

As shown in Table 2, univariate logistic regression analysis showed that age, sex, SBP, hypertension history, diabetes history, CHD history, smoking history, drinking history, SBP, CRP, FPG, HbA1c, TC, LDL‐C, homocysteine, eGFR, uric acid, and NLR were significantly associated with CSVD. Multivariate logistic regression analysis revealed that NLR (OR = 1.929, 95% CI = 1.599–2.327, *p* < .001) was independently associated with CSVD after adjusting for other confounding variables.

**Table 2:** Univariate and multivariate logistic regression analysis of factors affecting cerebral small vessel disease.

	Univariate analysis		Multivariable analysis	
	OR (95% CI)	*p*	OR (95% CI)	*p*
Sex (male)	2.271 (1.660–3.107)	<.001	‐	‐
Age	1.102 (1.083–1.121)	<.001	1.050 (1.023–1.079)	<.001
SBP	1.041 (1.032–1.048)	<.001	1.019 (1.007–1.030)	.001
Hypertension history	6.357 (4.513–8.955)	<.001	2.425 (1.466–4.011)	.001
Diabetes history	8.813 (4.487–17.311)	<.001	3.522 (1.304–9.516)	.013
CHD history	6.771 (2.630–17.430)	<.001	‐	‐
Smoking history	4.839 (3.300–7.096)	<.001	4.764 (2.457–9.236)	<.001
Drinking history	4.867 (2.682–8.830)	<.001	‐	‐
CRP	1.043 (1.012–1.075)	.006	‐	‐
FPG	1.437 (1.254–1.645)	<.001	‐	‐
HbA1c	2.196 (1.706–2.826)	<.001	‐	‐
TC	0.822 (0.727–0.930)	.002	1.576 (1.054–2.356)	.027
LDL‐C	0.706 (0.598–0.833)	<.001	0.521 (0.308–0.883)	.015
Homocysteine	1.163 (1.116–1.212)	<.001	1.056 (1.006–1.108)	.029
eGFR	0.961 (0.951–0.970)	<.001	‐	‐
Uric acid	1.003 (1.001–1.004)	<.001	‐	‐
NLR	2.605 (2.189–3.099)	<.001	1.929 (1.599–2.327)	<.001^a^

Abbreviations: CI, confidence interval; CRP, C‐reactive protein; DBP, diastolic blood pressure; eGFR, estimated glomerular filtration rate; FPG, fasting plasma glucose; HbA1c, hemoglobin A1c; LDL‐C, low‐density lipoprotein cholesterol; NLR, neutrophil/lymphocyte ratio; OR, odds ratio; SBP, systolic blood pressure; TC, total cholesterol; TG, triglyceride; WBC, white blood cell.

The symbol “‐” indicates that the variables were with *p* > .05.

^a^ Adjusted for age, sex, SBP, hypertension history, diabetes history, CHD history, smoking history, drinking history, CRP, FPG, HbA1c, TC, LDL‐C, homocysteine, eGFR, and uric acid.

### 3.3 Association of NLR with WMH burden

**Figure 1:**
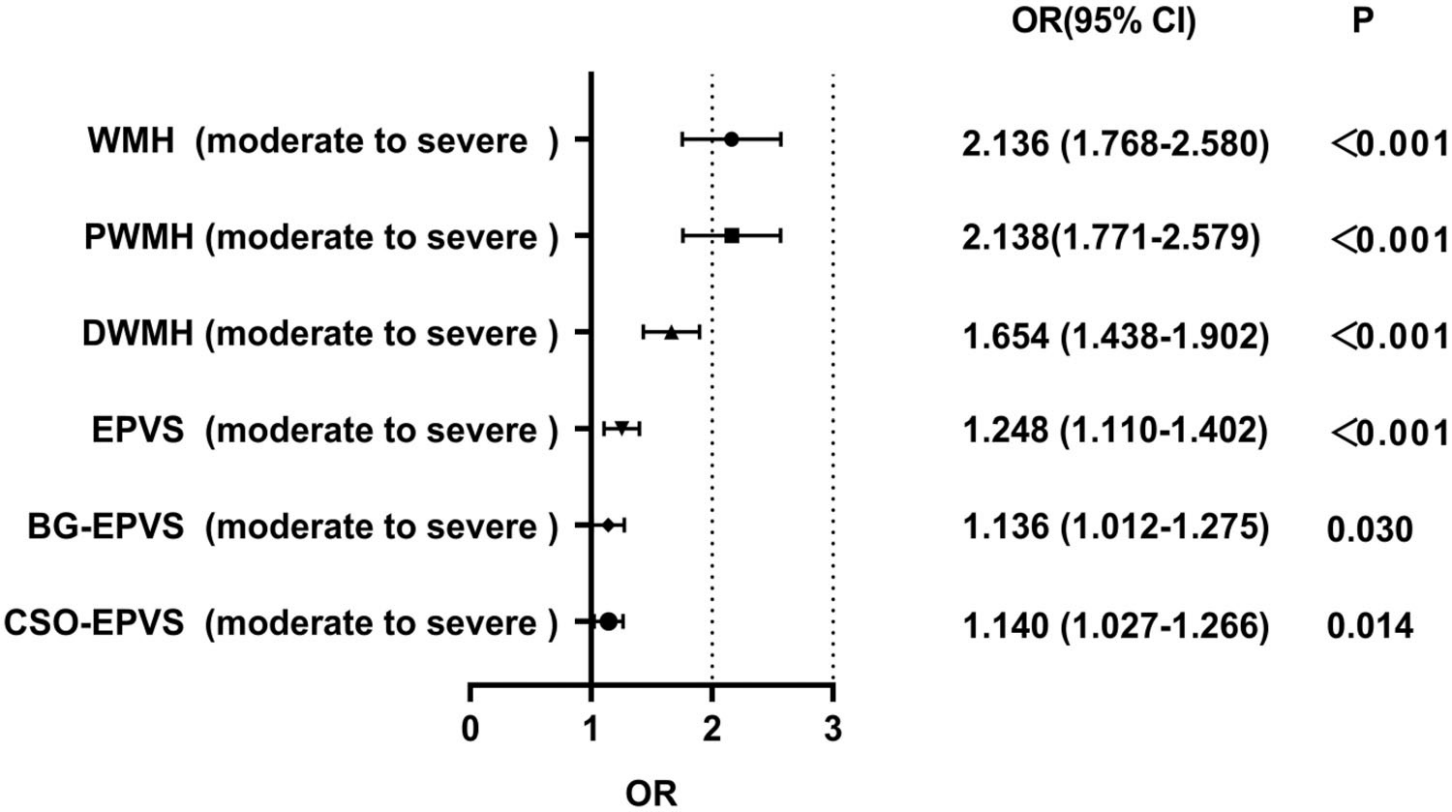
Forest plots for the association of NLR with the severity of WMH and EPVS. Adjusted for age, sex, body mass index, SBP, DBP, hypertension history, diabetes history, CHD history, smoking history, drinking history, CRP, FPG, HbA1c, TG, TC, LDL‐C, homocysteine, eGFR, and uric acid. BG‐EPVS, basal ganglia enlarged perivascular spaces; CSO‐EPVS, centrum semiovale enlarged perivascular spaces; DWMH, deep white matter hyperintensity; EPVS, enlarged perivascular spaces; NLR, neutrophil/lymphocyte ratio; PWMH, periventricular white matter hyperintensity; WHM, white matter hyperintensity.

Multivariate logistic regression analysis was performed to analyze the association of NLR with WMH severity. As shown in Table 3 and Figure 1, NLR (OR = 2.136, 95% CI = 1.768–2.580, *p* < .001) remained an independent risk factor for moderate to severe WMH after adjusting for other confounding variables. Furthermore, NLR was also independently associated with moderate to severe PWMH (OR = 2.138, 95% CI = 1.771–2.579, *p* < .001), and moderate to severe DWMH (OR = 1.654, 95% CI = 1.438–1.902, *p* < .001) (Table 3 and Figure 1).

**Table 3:** The logistic regression analysis for association between NLR and severity of WMH.

	Moderate to severe WMH (score of 3–6)		Moderate to severe PWMH (score of 2–3)		Moderate to severe DWMH (score of 2–3)	
	OR (95% CI)	*p*	OR (95% CI)	*p*	OR (95% CI)	*p*
Model 1^a^						
NLR	2.432 (2.023–2.923)	<.001	2.367 (1.980–2.829)	<.001	1.728 (1.513–1.973)	<.001
Model 2^b^						
NLR	2.136 (1.768–2.580)	<.001	2.138 (1.771–2.579)	<.001	1.654 (1.438–1.902)	<.001

Abbreviations: CI, confidence interval; DWMH, deep white matter hyperintensity; NLR, neutrophil/lymphocyte ratio; OR, odds ratio; PWMH, periventricular white matter hyperintensity; WMH, white matter hyperintensity.

^a^ Model 1: multivariable logistic regression model adjusted for age and sex.

^b^ Model 2: multivariable logistic regression model adjusted for age, sex, BMI, SBP, DBP, hypertension history, diabetes history, CHD history, smoking history, drinking history, CRP, FPG, HbA1c, TG, TC, LDL‐C, homocysteine, eGFR, and uric acid.

### 3.4 Association of NLR with EPVS burden

Multivariate logistic regression analysis of EPVS severity revealed that NLR (OR = 1.248, 95% CI = 1.110–1.402, *p* < .001) remained an independent risk factor for moderate to severe EPVS after adjusting for other confounding variables (Table 4 and Figure 1). Moreover, NLR was also independently associated with moderate to severe BG‐EPVS (OR = 1.136, 95% CI = 1.012–1.275, *p* = .030) and moderate to severe CSO‐EPVS (OR = 1.140, 95% CI = 1.027–1.266, *p* = .014) (Table 4 and Figure 1).

**Table 4:** The logistic regression analysis for association between NLR and severity of EPVS.

	Moderate to severe EPVS (score of 2–4)		Moderate to severe BG‐EPVS (score of 2–4)		Moderate to severe CSO‐EPVS (score of 2–4)	
	OR (95% CI)	*p*	OR (95% CI)	*p*	OR (95% CI)	*p*
Model 1^a^						
NLR	1.348 (1.206–1.505)	<.001	1.188 (1.069–1.322)	.001	1.197 (1.087–1.318)	<.001
Model 2^b^						
NLR	1.248 (1.110–1.402)	<.001	1.136 (1.012–1.275)	.030	1.140 (1.027–1.266)	.014

Abbreviations: BG‐EPVS, basal ganglia enlarged perivascular spaces; CI, confidence interval; CSO‐EPVS, centrum semiovale enlarged perivascular spaces; EPVS, enlarged perivascular spaces; NLR, neutrophil/lymphocyte ratio; OR, odds ratio.

^a^ Model 1: multivariable logistic regression model adjusted for age and sex.

^b^ Model 2: multivariable logistic regression model adjusted for age, sex, BMI, SBP, DBP, hypertension history, diabetes history, CHD history, smoking history, drinking history, CRP, FPG, HbA1c, TG, TC, LDL‐C, homocysteine, eGFR, and uric acid.

### 3.5 Logistic regression analysis of NLR and lacune

As shown in Table 5, univariate logistic regression analysis found that age, sex, SBP, hypertension history, diabetes history, CHD history, smoking history, drinking history, CRP, FPG, HbA1c, TC, LDL‐C, homocysteine, eGFR, uric acid, and, NLR were significantly related with Lacune. Further analysis with multivariate logistic regression revealed that age (OR = 1.039, 95% CI = 1.015–1.064, *p* = .001), hypertension history (OR = 2.367, 95% CI = 1.487–3.768; *p* < .001), smoking history (OR = 2.924, 95% CI = 1.684–5.074, *p* < .001), SBP (OR = 1.011, 95% CI = 1.002–1.020, *p* = .016), and homocysteine (OR = 1.027, 95% CI = 1.007–1.048; *p* = .009) were independently associated with Lacune (Table 5). However, there was no significant association of NLR with Lacune after adjustment for other confounders (Table 5).

**Table 5:** The logistic regression analysis for the association between NLR and lacune.

	Univariate analysis		Multivariable analysis	
	OR (95% CI)	*p*	OR (95% CI)	*p*
Sex (male)	2.488 (1.677–3.691)	<.001	‐	‐
Age	1.060 (1.042–1.079)	<.001	1.039 (1.015–1.064)	.001
SBP	1.025 (1.018–1.033)	<.001	1.011 (1.002–1.020)	.016
Hypertension history	4.376 (2.931–6.535)	<.001	2.367 (1.487–3.768)	<.001
Diabetes history	2.156 (1.359–3.422)	.001	‐	‐
CHD history	2.841 (1.511–5.340)	.001	‐	‐
Smoking history	3.569 (2.454–5.191)	<.001	2.924 (1.684–5.074)	<.001
Drinking history	2.540 (1.575–4.096)	<.001	‐	‐
FPG	1.108 (1.016–1.209)	.021	‐	‐
HbA1c	1.274 (1.104–1.469)	.001	‐	‐
TC	0.842 (0.728–0.974)	.021	‐	‐
LDL‐C	0.757 (0.622–0.921)	.005	‐	‐
Homocysteine	1.045 (1.022–1.068)	<.001	1.027 (1.007–1.048)	.009
eGFR	0.984 (0.975–0.992)	<.001	‐	‐
Uric acid	1.002 (1.000–1.004)	.013	‐	‐
NLR	1.309 (1.194–1.435)	<.001	‐	‐a

Abbreviations: CI, confidence interval; CRP, C‐reactive protein; DBP, diastolic blood pressure; eGFR, estimated glomerular filtration rate; FPG, fasting plasma glucose; HbA1c, hemoglobin A1c; LDL‐C, low‐density lipoprotein cholesterol; NLR, neutrophil/lymphocyte ratio; OR, odds ratio; SBP, systolic blood pressure; TG, triglyceride; TC, total cholesterol; WBC, white blood cell.

The symbol “‐” indicates that the variables were with *p* > .05.

^a^ Adjusted for age, sex, SBP, hypertension history, diabetes history, CHD history, smoking history, drinking history, CRP, FPG, HbA1c, TC, LDL‐C, homocysteine, eGFR, and, uric acid.

### 3.6 The predictive ability of NLR and other risk factors for CSVD

**Figure 2:**
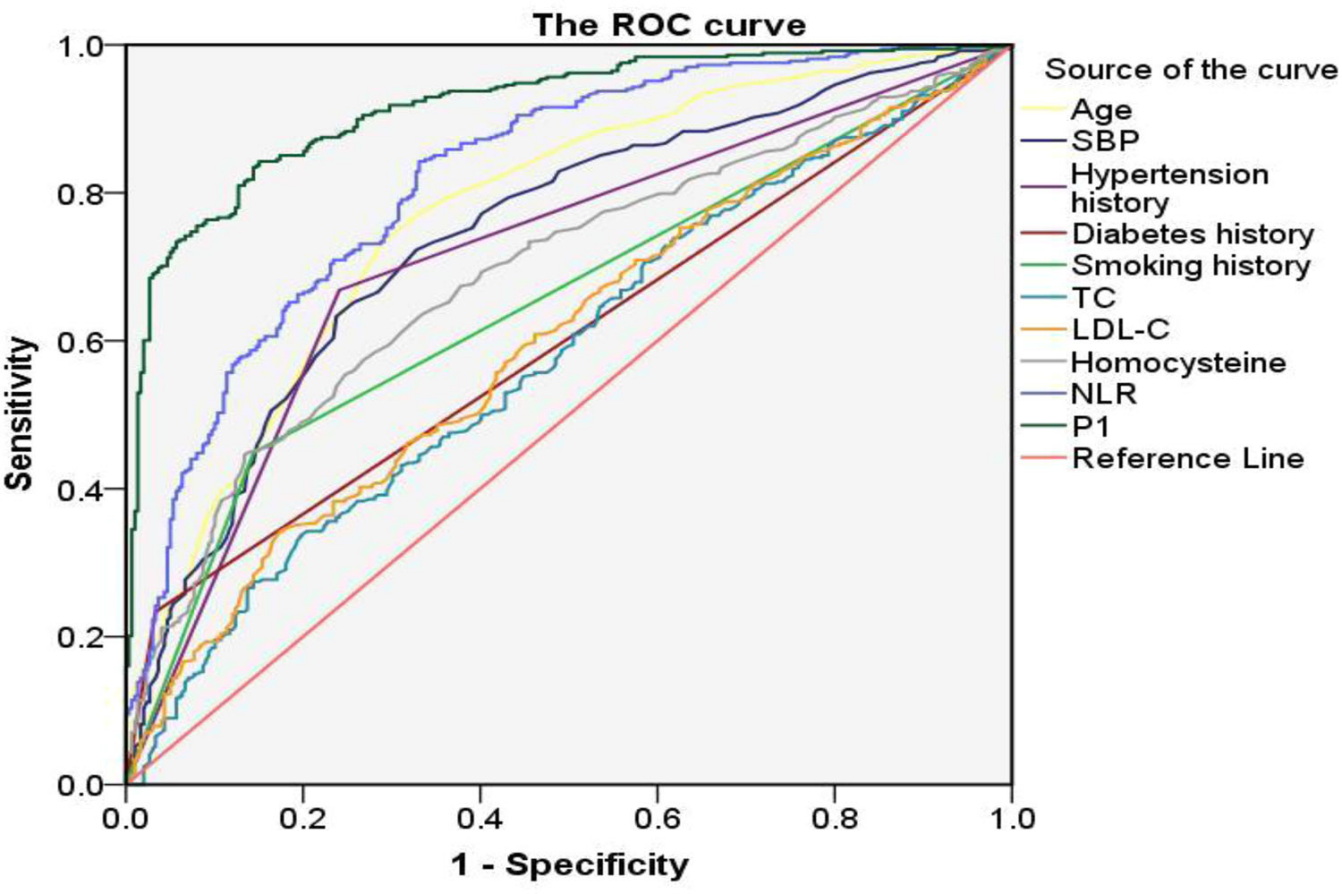
Receiver operating characteristic curve analysis for predicting cerebral small vessel disease (CSVD). LDL‐C, low‐density lipoprotein cholesterol; NLR, neutrophil/lymphocyte ratio; P1, the probability of predicting CSVD by combining NLR with age, SBP, hypertension history, diabetes history, smoking history, TC, LDL‐C, and homocysteine; SBP, systolic blood pressure; TC, total cholesterol.

We performed ROC curve analysis to determine the predictive ability of NLR and other risk factors for CSVD. The results showed that the area under the curve (AUC) of NLR for predicting CSVD was 0.822 (95% CI = 0.790–0.854, *p* < .01) and the optimal cutoff value of NLR was 2.47 (Figure 2). When NLR was at the optimal cutoff value, the sensitivity for predicting CSVD was 84.2% and the specificity was 66.9%. The probability (P1) of predicting CSVD by combining NLR with age, SBP, hypertension history, diabetes history, smoking history, TC, LDL‐C, and homocysteine was calculated using binary logistic regression. The AUC of P1 was 0.916 (95% CI = 0.896–0.937, *p* < .01). The optimal cutoff value for P1 was 0.516, with a sensitivity of 84.2% and a specificity of 84.9% (Figure 2) (Table 6). These results reveal a higher predictive effect for NLR compared to other risk factors, while the diagnostic effect is maximized when NLR is combined with other risk factors.

**Table 6:** Receiver operating characteristic curve analysis for predicting cerebral small vessel disease.

	AUC	95% CI	*p*
Age	0.771	0.735–0.807	<.001
SBP	0.741	0.704–0.779	<.001
Hypertension history	0.714	0.674–0.753	<.001
Diabetes history	0.600	0.558–0.643	<.001
Smoking history	0.652	0.611–0.694	<.001
TC	0.581	0.538–0.624	<.001
LDL‐C	0.598	0.556–0.641	<.001
Homocysteine	0.692	0.652–0.732	<.001
NLR	0.822	0.790–0.854	<.001
P1	0.916	0.896–0.937	<.001

Abbreviations: AUC, area under the curve; CI, confidence interval; LDL‐C, low‐density lipoprotein cholesterol; NLR, neutrophil/lymphocyte ratio; P1, the probability of predicting cerebral small vessel disease by combining NLR with age, SBP, hypertension history, diabetes history, smoking history, TC, LDL‐C, and homocysteine; SBP, systolic blood pressure; TC, total cholesterol.

## 4 DISCUSSION

In the present study, we found that NLR was significantly associated with CSVD, which was consistent with a previous study.^33^ It is speculated that the mechanism may be similar to the role of inflammatory cells in acute ischemic stroke. Previous studies have confirmed that when the brain tissue is under hypoxic‐ischemic conditions, the circulating leukocytes may be activated, and the blood‐brain barrier may be impaired, thus aggravating neuronal damage.^34^, ^35^, ^36^, ^37^ Meanwhile, the inflammatory mediators may be released into the blood, triggering a series of inflammatory reactions.^38^ In addition, it has been suggested that cerebral ischemia may decrease the number of circulating immune cells by activating the sympathetic nervous system and the hypothalamic‐pituitary‐adrenal axis, resulting in a state of immunosuppression.^39^ Furthermore, our study showed that the CSVD group had a higher NLR than the non‐CSVD group, which further supports the inflammatory mechanism of CSVD.

We also found that NLR was independently associated with moderate to severe WMH after adjusting for confounding factors. This is consistent with previous findings.^11^, ^18^ However, the relationship of NLR with PWMH and DWMH has not been reported. Here, our results showed that NLR was independently associated with moderate to severe PWMH and moderate to severe DWMH after adjusting for confounders. The mechanisms underlying the relationship between NLR and WMH are not clear, and we have put forward several possible explanations. First, endothelial dysfunction may be a possible explanation. Normal endothelial cells can secrete vasodilators (e.g., nitric oxide, prostacyclin) and antithrombotic agents.^40^ Under chronic inflammation, activated leukocytes (particularly neutrophils) increase their ability to adhere to vascular endothelium, thereby deregulating endothelial cell regulation and ultimately leading to blood‐brain barrier disruption.^11^, ^41^ Impairment of the blood‐brain barrier allows the release of various toxic metabolites into the periventricular space and damages the surrounding neural tissues, thus inducing pathological changes in white matter regions and leading to WMH. Second, atherosclerosis may be another possible explanation. It is well‐known that chronic systemic inflammation is an important mechanism leading to atherosclerosis.^42^ As demonstrated by Balta et al., higher NLR was strongly associated with atherosclerosis.^43^ Intracranial and extracranial atherosclerosis can lead to diffuse hypoperfusion of the brain, decrease of cerebral blood flow, and damage of the white matter of the brain.^44^ Finally, this may be a combined effect of many vascular risk factors. In previous studies, chronic inflammation has been associated with various vascular risk factors, including diabetes, hypertension, smoking, obesity, and metabolic syndrome,^9^, ^19^ which are all strongly associated with the development of WMH.

Meanwhile, after multivariate logistic regression analysis, NLR was found to be independently associated with moderate to severe EPVS in this study. Consistently, Wang et al also demonstrated a positive trend between NLR and the number of EPVS.^33^ In a community‐based study by Jiang et al, NLR was found to be independently associated with moderate to severe BG‐EPVS.^45^ However, they did not study CSO‐EPVS. In this study, we further investigated the relationship of NLR with BG‐EPVS and CSO‐EPVS. The results showed that NLR was independently associated with moderate to severe BG‐EPVS and moderate to severe CSO‐EPVS. This may be explained by the following reasons. First, after ischemia or injury of the brain tissue, activated inflammatory cells accumulate around the blood vessels, and chemokines and cytokines are released to degrade the extracellular matrix, resulting in increased permeability of the blood‐brain barrier, leakage of blood components into the perivascular space, and promoting EPVS formation.^46^ Second, when the body is in an inflammatory state, inflammatory cells will accumulate excessively, obstructing the perivascular space and abnormal accumulation of cerebrospinal fluid, harmful substances, and β‐amyloid, as well as resulting in perivascular space expansion.^8^, ^47^

In addition, we found no significant association between NLR and Lacune. This is in agreement with the results by Jiang et al.^45^ However, previous study has also shown that higher NLR was associated with silent cerebral infarction.^11^ This difference may be due to differences in the study population, ethnicity, or insufficient sample size. In the future, it is necessary to expand the sample size and perform a multicenter study to verify the relationship between NLR and Lacune.

Finally, ROC analysis showed that the AUC was 0.822 for NLR to predict CSVD, the optimal cutoff point was 2.47, the sensitivity was 84.2%, and the specificity was 66.9%. This result indicates a good predictive value of NLR for CSVD. Furthermore, the diagnostic effect was the best when NLR was combined with other risk factors.

The strengths of this study are as follows. First, this study is the first to report the relationship of NLR with PWMH, DWMH, BG‐EPVS, and CSO‐EPVS, providing new evidence for the involvement of inflammation in the pathogenesis of CSVD. Second, NLR can be easily acquired at a low cost and its use in evaluating the prevalence and progression of CSVD holds promising clinical applicability.

There are some limitations in this study. First, this study is a single‐center, retrospective, cross‐sectional data analysis. There may be selection bias, and the causal relationship cannot be established. Further prospective studies are needed to confirm such findings. Second, the sample size is relatively small. Thus, it is necessary to expand the sample size in the future to further confirm the study results. Third, neutrophil and lymphocyte counts have temporal variability. This study only discussed the relationship between CSVD and the NLR values measured for the first time after admission. No dynamic observation was performed. Finally, the relationship of NLR with other common imaging markers of CSVD was not analyzed, which needs to be further explored.

## 5 CONCLUSIONS

In this study, we found NLR was significantly related to CSVD and common imaging markers, including WMH, PWMH, DWMH, EPVS, BG‐EPVS, and CSO‐EPVS. NLR may serve as a valid and convenient biomarker for assessing CSVD.

## AUTHOR CONTRIBUTIONS

Jiangping Cai and Xiaoyi Zeng performed the research. Xiaolan Wei designed the research study. Xiaojin Huang and Hansheng Dong contributed essential reagents or tools. Junyi Liu, Jie Lin, and Meirong Xie analyzed the data. Jiangping Cai, Xiaoyi Zeng, and Xiaolan Wei wrote the paper. All authors have read and approved the final manuscript.

## CONFLICT OF INTEREST STATEMENT

The authors declare no conflict of interest.

## ETHICS STATEMENT

All methods were carried out following the Declaration of Helsinki. This study was approved by the Ethics Committee of Quanzhou First Affiliated Hospital of Fujian Medical University. Written informed consent was obtained from each participant.

## Data Availability

The data that support the findings of this study are available from the corresponding author upon reasonable request.
